# A case of prosthetic joint infection due to the rare opportunist yeast, *Cyberlindnera fabianii*

**DOI:** 10.22034/CMM.2023.345063.1418

**Published:** 2023-09

**Authors:** Nurhafiza Ishak, Kallaivani Pachayappan, Chu Lee Hwa, Muhammad Asyraf Mohamad Kamil

**Affiliations:** 1 Microbiology Unit, Department of Pathology, Hospital Kuala Lumpur, Malaysia; 2 Bacteriology Unit, Infectious Disease Research Centre, National Institutes of Health, Malaysia

**Keywords:** *Cy. Fabianii*, Invasive fungal, Prosthetic joint infection

## Abstract

**Background and Purpose:**

Invasive fungal infections caused by *Cyberlindnera fabianii* (*Cy. fabianii*) have recently increased despite the low virulence potential of this yeast. However, limited information about the widely-used commercial biochemical identification systems has led to an underestimation of *Cy. fabianii* infections in clinical settings.

**Case report:**

This study reports a case of prosthetic joint infection in a patient who had a previous history of total knee replacement surgery. *Cy. fabianii* was recovered from intraoperative culture specimens identified by matrix-assisted laser desorption/ionization time-of-flight mass spectrometry and confirmed using molecular assays. It was, however, initially misidentified as *Candida utilis* by phenotypic identification.

**Conclusion:**

Due to the emergence of uncommon yeast species, it is important to accurately identify and perform antifungal susceptibility testing on uncommon yeast isolates for appropriate management.

## Introduction

*Cyberlindnera fabianii* (*Cy. fabianii*) is an uncommon yeast that rarely causes invasive fungal infections in humans [ 1 ]. A review of the literature has shown that despite its low virulence potential, *Cy. fabianii* has been isolated from various clinical specimens, such as blood, urine, sputum, stool, peritoneal fluid, pleural fluid, and cerebrospinal fluid [ 1 - 3 ]. 

Herein, this study describes the first case report of a late-onset prosthetic joint infection (PJI) due to *Cy. fabianii* initially misidentified as *Candida* species by a commercial biochemical system. *Cy. fabianii* appears to be an opportunistic agent that can lead to significant morbidity and mortality [ 2 ]. The advancement of identification testing using matrix-assisted laser desorption/ionization time-of-flight mass spectrometry (MALDI-TOF) and molecular assays is crucial for identifying organisms accurately and ensuring appropriate diagnosis and management.

## Case report

**Figure 1:**
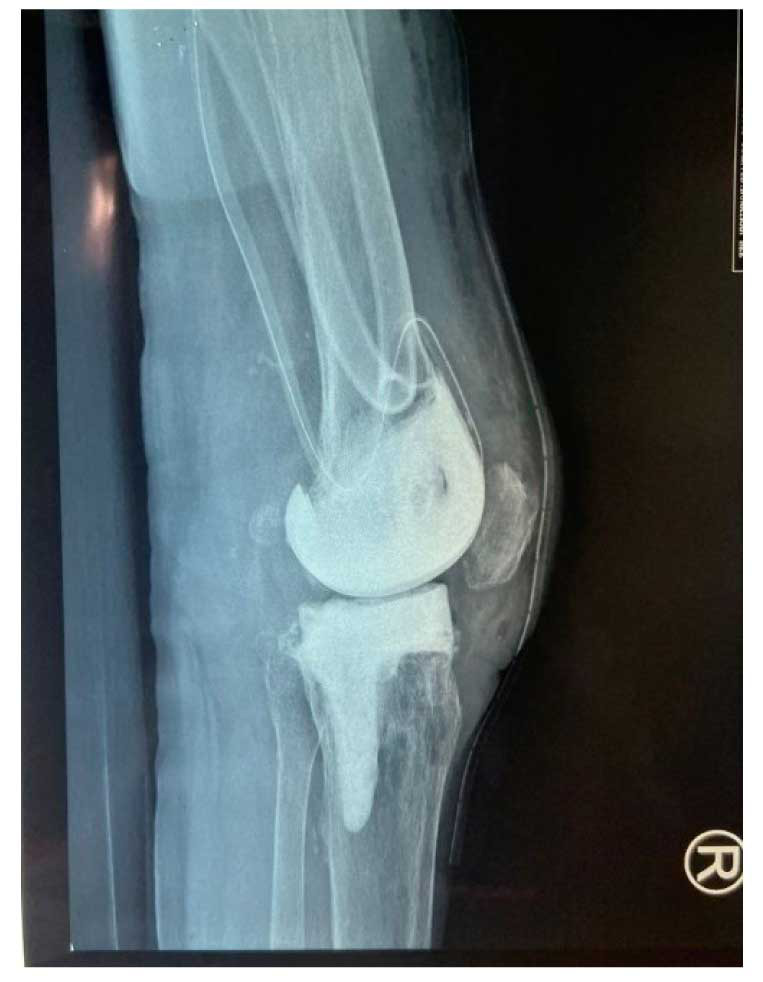
Radiology examination of the right knee

An 85-year-old Chinese woman with underlying hypertension, dyslipidemia, and a previous history of right total knee replacement surgery seven years earlier presented with painful right knee swelling, accompanied by pus discharge, for three months. She denied any recent trauma or procedures. On admission, she was afebrile, and her right knee appeared diffusely swollen, erythematous, and tender with a sinus tract formation. The total white blood cell count was 7.8×10^3^ cells/µL with an elevated C-reactive protein and erythrocyte sedimentation rate. Radiology findings revealed radiolucency over the distal femur and proximal tibia (Figure 1). She was diagnosed with right knee PJI and osteomyelitis. Therefore, intravenous clindamycin and ciprofloxacin were started as empirical therapies. She underwent the removal of the implant and an arthrotomy washout. The intraoperative findings were suggestive of osteomyelitis changes.

**Figure 2:**
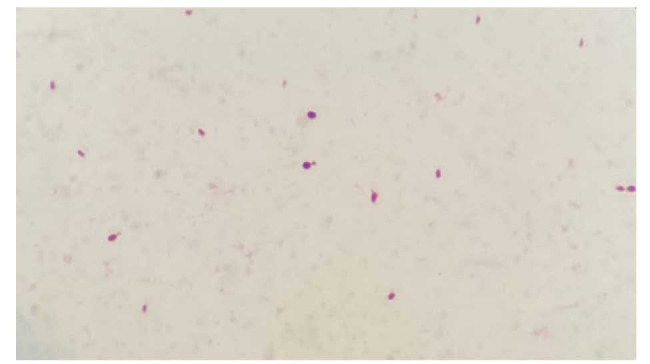
Budding yeast cells in synovial fluid microscopic examination at 1000× magnification

**Figure 3:**
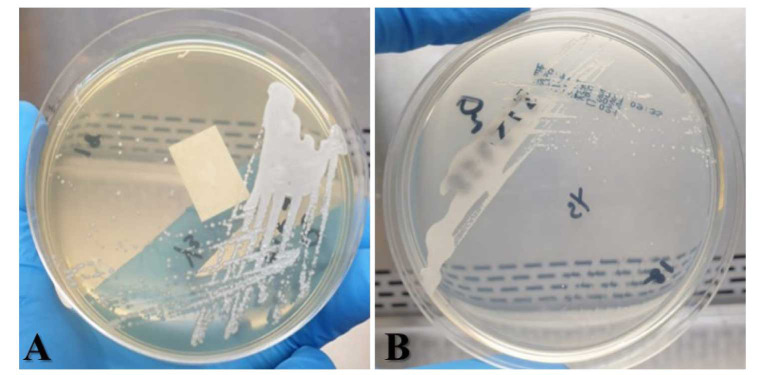
**a and 3b:** Cy. fabianii colonies on the Sabouraud Dextrose Agar and the CHROMagar

The intraoperative synovial fluid was inoculated in BD Bactec Plus Aerobic/F and BD Bactec Lytic/10 Anaerobic/F vials, which were incubated using BD BACTEC^TM^ FX (BD, USA). The aerobic bottle blood culture was positive after 72 h of incubation. The gram stain showed gram-positive ellipsoidal budding yeast cells (Figure 2), and the organism appeared as cream-colored smooth, glistening colonies on the Sabouraud Dextrose Agar (Isolab, Malaysia) and whitish colonies on the CHROMagar (Isolab, Malaysia), as displayed in Figures 3a and 3b, respectively. 

The slide culture on the corn meal agar only demonstrated blastoconidia, and it was negative for both germ tube and urease. The same growth was also obtained from bone marrow specimens. The organism was identified as *Candida utilis* (*C. utilis*) with a probability of 95% using the VITEK® 2 yeast identification system (bioMérieux, France). However, a final microbiology report revealed that *Cy. fabianii* was identified by Bruker MALDI-TOF, which was also confirmed by molecular DNA sequencing methods. 

The DNA extraction of the isolate was performed using the Quick-DNA Fungal/Bacterial Miniprep Kit (Zymo Research, California, USA), following the manufacturer’s protocol. The D1/D2 domain of the large subunit (LSU) ribosomal RNA gene was amplified from the DNA template by NL1 and NL4 primers. Polymerase Chain Reaction (PCR) was performed using Mastercycler Gradient (Eppendorf, Hamburg, Germany). The PCR protocol was conducted with an initial denaturation at 95°C for 1 min, followed by 35 cycles of denaturation (95°C for 15 sec), annealing (58°C for 15 sec), and extension (72°C for 10 sec) [ 4 ]. The PCR product was then visualized using a 1.5% agarose gel by electrophoresis. The sample was then sent to the 1^st^ BASE company (Apical Scientific, Malaysia) for PCR purification and sequencing. 

**Figure 4:**
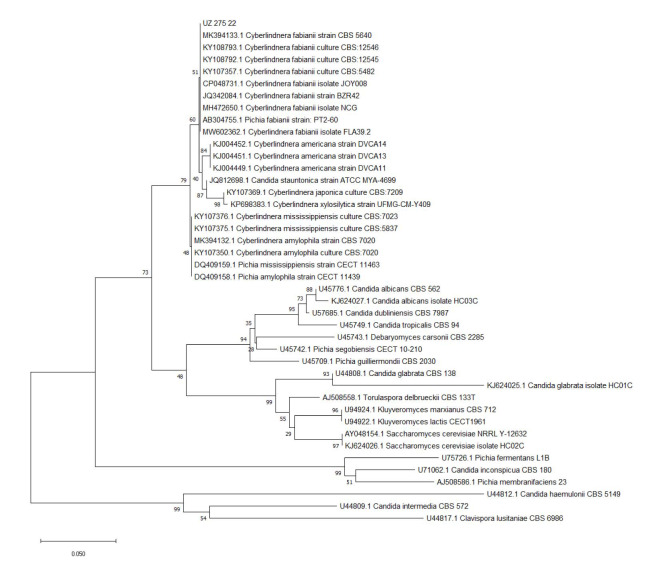
Phylogenetic tree inferred from neighbor-joining analysis of the D1/D2 region of the large subunit sequences of *Cy. fabianii* species

Sequencing analysis was performed using Molecular Evolutionary Genetics Analysis (MEGA) software (version 11.0.10, Arizona, USA), and alignment was made to both forward and reverse sequencing results using the MUSCLE algorithm. The 595 DNA bp was subsequently matched to the database from the National Center for Biotechnology Information (NCBI) GenBank using the Basic Local Alignment Search Tool (BLAST). The result was a 100% match to *Cy. fabianii* CBS 5640 (GenBank Accession Number KY107357). For phylogenetic analyses, the D1/D2 region of the LSU rRNA gene was constructed using the MEGA software, and phylogenetic trees were generated by the neighbor-joining statistical method with 1000 bootstrap replications using the Tamura-Nei substitution method (Figure 4). All available LSU sequences closely related to this species and other distant yeast species were retrieved from the NCBI GenBank database. 

Antifungal susceptibility testing was performed using VITEK® 2 AST-YS08 (bioMérieux, France), and the minimum inhibitory concentration (MIC) values were interpreted based on Clinical and Laboratory Standards Institute M60 interpretative breakpoints for *Candida* species [ 5 ]. It was susceptible to amphotericin B (MIC<0.12 µg/ml), fluconazole (MIC<0.5 µg/ml), voriconazole (MIC<0.12 µg/ml), and micafungin (MIC<0.25 µg/ml). No growth was obtained from blood and tissue cultures. The patient completed intravenous fluconazole for two weeks and was discharged with oral fluconazole and ampicillin-sulbactam planned for a total of 12 and 10 weeks, respectively. 

## Discussion

PJI is a serious complication of joint replacement surgery and poses a significant challenge as it requires prolonged hospitalization and surgery. The diagnosis of PJI is not always straightforward, which may result in delayed management. 

*Cy. fabianii* has been associated with fungemia, endocarditis, pneumonia, prostatitis, peritonitis, meningitis, and catheter-related infections with variable outcomes [ 6 - 8 ]. Arastehfar et al. have presented a comprehensive review of 39 *Cy. fabianii* infection cases, along with clinical information, risk factors, and microbiological findings [ 8 ]. To the best of the researchers’ knowledge, this is the first case report of a late-onset PJI caused by *Cy. fabianii*. Fungus and mycobacterium have minor contributions to PJI cases, and the diagnosis may be difficult as isolation and identification may take a longer time [ 9 - 10 ].

The development of PJI due to *Candida* species may be associated with biofilm formation on prostheses, as well as virulence factors, such as adherence and hydrolytic enzyme secretions [ 11 ]. Direct inoculation during surgery or procedures, extension from infected surrounding tissues, and a hematogenous route from distant sources of infection are the possible routes of PJI [ 12 ]. However, in this case report, the source of infection was undetermined because no growth was obtained from blood culture or intraoperative tissue specimens.

*Cy. fabianii* is an environmental yeast present in soil. It is an ascomycetous yeast of the Saccharomycetaceae family, which was formerly known as *Hansenula fabianii*, *Pichia fabianii*, and *Lindnera fabianii*. Later, however, the new genus Cyberlindnera was proposed as an alternative name [ 13 - 14 ]. There was no information about *Cy. fabianii* in the database of commercial biochemical systems, and thus it has been misidentified as *C. pelliculosa*, *C. utilis*, *Cy. jadinii*, or *Wickerhamomyces anomalus* by the Analytical Profile Index and the VITEK system [ 8 ],[ 15 ]. Molecular testing and MALDI-TOF have been promising tools and reliable methods for the identification of rare yeast [ 1 ],[ 16 ]. 

For phylogenetic analyses, all available LSU sequences closely related to this species were retrieved from the NCBI GenBank database. The LSU sequence of *Cy. fabianii* was used as an outgroup taxon. The isolate in this case was identical to *Cy. fabianii* type strain 5640, which was genetically closely related to the isolates from three clustered cases of the pseudo-outbreak of *Cy. fabianii* in a tertiary hospital in Beijing, China [ 17 ]. 

The misidentification of organisms may influence precision in clinical recognition, which can lead to inappropriate patient management because uncommon yeast, such as *Cy. Fabianii*, may exhibit decreased susceptibility to commonly used antifungal agents.

The emergence of antifungal resistance and the ability of *Cy. fabianii* to cause severe, life-threatening, invasive infections have reinforced accurate identification for proper patient management [ 18 ]. The potential outcome of misdiagnosis and inappropriate treatment of PJI can result in the persistence of infection with deleterious consequences, including disability and impaired quality of life.

Fungal PJIs represent a therapeutic challenge as they may require prolonged antifungal treatment. Amphotericin B, azole group, flucytosine, and echinocandins exhibited good antifungal activities against the majority of *Cy. fabianii* isolates [ 1 ]. However, a potential to develop resistance to amphotericin B, fluconazole, itraconazole, and voriconazole has been observed in a few studies [ 8 ], [ 19 - 20 ]. 

*Cy. fabianii* can form a biofilm, allowing it to resist azole group antifungals [ 20 ]. Therefore, antifungal susceptibility testing should be pursued as strains of this yeast can have varied MIC values. The optimal treatment option will be a combination of surgical removal of the prosthetic devices and prolonged antifungal therapy. In this case report, the patient was successfully treated with parenteral fluconazole for two weeks, followed by oral fluconazole for at least 12 weeks. 

## Conclusion

Due to the emergence of uncommon yeast species causing opportunistic infections and the limitations of conventional identification methods, more sensitive and specific advanced approaches, such as MALDI-TOF and molecular assays, are crucial for the correct identification and epidemiological surveillance of such potential pathogens. Antifungal susceptibility testing is also essential for proper patient management.
